# Tetrandrine, an agonist of aryl hydrocarbon receptor, reciprocally modulates the activities of STAT3 and STAT5 to suppress Th17 cell differentiation

**DOI:** 10.1111/jcmm.13141

**Published:** 2017-03-22

**Authors:** Xusheng Yuan, Yannong Dou, Xin Wu, Zhifeng Wei, Yue Dai

**Affiliations:** ^1^ Department of Pharmacology of Chinese Materia Medica China Pharmaceutical University Nanjing China

**Keywords:** tetrandrine, Th17 cell differentiation, aryl hydrocarbon receptor, STAT3, STAT5

## Abstract

Tetrandrine, a bisbenzylisoquinoline alkaloid constituent of the root of *Stephania tetrandra* S. Moore, was previously shown to suppress the differentiation of T helper 17 (Th17) cells and consequently ameliorate the collagen‐induced arthritis (CIA) in mice by activating the aryl hydrocarbon receptor (AhR), but its underlying mechanism is incompletely understood. Here, we investigated how tetrandrine suppressed Th17 cell differentiation through the AhR pathway. The naïve CD4^+^ T cells were stimulated with anti‐CD3/CD28 for 72 hrs in the presence or absence of tetrandrine under the Th17‐polarizing condition. Tetrandrine inhibited the phosphorylation of signal transducer and activator of transcription‐3 (STAT3) and boosted the phosphorylation of STAT5, while it did not alter the expression levels of phospho‐Janus kinase‐1 (p‐JAK1), p‐JAK2, p‐JAK3, and suppressor of cytokine signalling‐3 (SOCS3). The tetrandrine‐mediated inhibition of the Th17 cell differentiation could be diminished by the activator of STAT3 and the inhibitor of STAT5. Meanwhile, the effect of tetrandrine on the either STAT3 or STAT5 phosphorylation was almost completely reversed by the AhR antagonist CH223191 and the AhR knockdown. In CIA mice, tetrandrine decreased p‐STAT3 levels and increased p‐STAT5 levels, which could also be reversed by the AhR antagonist resveratrol administration. Furthermore, tetrandrine promoted the AhR binding to the STAT5, but not to the STAT3. The tetrandrine‐induced inhibition of the STAT3 phosphorylation was diminished by the inhibitor of STAT5. Taken together, tetrandrine suppressed Th17 cell differentiation by reciprocally modulating the activities of STAT3 and STAT5 in an AhR‐dependent manner.

## Introduction

Tetrandrine possesses a therapeutic efficacy for rheumatic diseases 1, 2, 3. Recently, we demonstrated that tetrandrine could ameliorate the collagen‐induced arthritis (CIA) in mice by suppressing the differentiation of T helper 17 (Th17) cells *via* the activation of aryl hydrocarbon receptor (AhR) 4. AhR is a ligand‐dependent transcription factor which belongs to the basic helix‐loop‐helix and Per‐ARNT‐Sim family and responds to the xenobiotics, most notably to 2,3,7,8‐tetrachlorodibenzo‐*p*‐dioxin (TCDD) 5, 6. It displays a dual role as an activator for the metabolism of molecules and as a player in many cell functions because it has promiscuous binding sites for the various ligands 7, 8. TCDD, a well‐established environmental contaminant, can induce the functional activation of AhR to inhibit the activation, proliferation and differentiation of T cells 9, 10. It was also able to suppress the differentiation of Th17 cells and the expressions of the transcription factor RORγt and the cytokine IL‐17 through the activation of AhR 11, 12, 13. However, the detailed mechanism by which tetrandrine and TCDD suppress Th17 cell differentiation *via* the activation of AhR remains unclear.

Accumulative evidence has shown that the Janus kinase (JAK) and signal transducer and activator of transcription (STAT) signalling pathways are essential for the differentiation of Th17 cells 14. The RORγt expression required for this process is initiated by the transforming growth factor‐β (TGF‐β) in combination with IL‐6, IL‐21, and IL‐23, all of them are closely related to the STAT3 15. The STAT3 phosphorylation also positively correlates with Th17 cell expansion and synovitis in the patients with rheumatoid arthritis (RA) 16. In contrast, the STAT5 which is functionally activated by phosphorylation can inhibit STAT3 activity and Th17 cell response 17, 18, 19. A significant deficiency of the STAT5 phosphorylation has been reported in the peripheral blood mononuclear cells from the patients with RA 20.

Notably, AhR is able to interact with the proteins of STAT5 and STAT1 by forming a complex and enhances their phosphorylations 21. The natural agonists of AhR have been shown to promote STAT5 activity for the inhibition of the Th17 cell differentiation 22. Tetrandrine, an agonist of AhR, was reported to attenuate the activity of STAT3 and thereby suppress the survival and proliferation of human neuroglioma cells 23. These findings suggest that STAT proteins might be involved in the tetrandrine‐mediated inhibition of Th17 cell differentiation through the AhR pathway. The purpose of this study was to investigate the relevance between the activation of AhR, modulation of STAT3 and STAT5 activities, and inhibition of Th17 cell differentiation caused by the tetrandrine treatment under the Th17‐polarizing condition, and gain insight into the underlying mechanism by which tetrandrine facilitates the interaction of AhR with STAT3 or STAT5. TCDD was used as a reference, and a comparative analysis with tetrandrine was conducted.

## Materials and methods

### Animals

The C57BL/6 mice (6–8 weeks) were purchased from the Comparative Medicine Centre of the Yangzhou University (Yangzhou, China). The male DBA/1 mice (6–8 weeks) were purchased from the Laboratory Animal Unit, Academy of the Military Medical Sciences (Beijing, China). The mice were housed in specific pathogen‐free facilities and were maintained at 25°C on a 12 hrs/12 hrs dark cycle with food and water available optionally. All of experimental procedures were performed according to the guidelines of the National Institutes of Health Guidelines for the Care and Use of Laboratory Animals.

### Chemicals

Tetrandrine (purity: more than 98%) was purchased from the Zelang Pharmaceutical Technology Co., Ltd (Nanjing, China), which was dissolved in 0.1 N HCl and was adjusted to pH 7.2 with 1 N NaOH. It was filtrated with nitrocellulose filter (Millipore, MA, USA) and was diluted to 10 mM stock solution. The TCDD (purity: 99.9%) was purchased from the AccuStandard, Inc. (AccuStandard, CT, USA). The AhR antagonist resveratrol was also purchased from the Zelang Pharmaceutical Technology Co., Ltd (Nanjing, China). 1‐methyl‐*N*‐[2‐methyl‐4‐[2‐(2‐methylphenyl)diazenyl]phenyl‐1H‐pyrazole‐5‐carboxamide (CH‐223191, an antagonist of AhR) was purchased from the Sigma‐Aldrich (St. Louis, MO, USA). The colivelin (an activator of STAT3) was purchased from the Abgent, Inc. (Abgent, San Diego, CA, USA). 2‐[(4‐oxo‐4H‐benzopyran‐3‐yl)methylene]hydrazide‐3‐pyridinecarboxylic acid (HPA, an inhibitor of STAT5) was purchased from the Cayman Chemical Co. (Cayman, MI, USA). The cryptotanshinone (CPT, an inhibitor of STAT3) was purchased from the MedChem Express LLC (MCE, NU, USA). The mouse antibodies against JAK1, phospho‐JAK1 (p‐JAK1), JAK2, p‐JAK2, JAK3, p‐JAK3, STAT1, p‐STAT1, STAT3, p‐STAT3, STAT5, p‐STAT5, suppressor of cytokine signalling‐3 (SOCS3), AhR, and cytochrome P450 1A1 (CYP1A1) were purchased from the Santa Cruz Biotechnology, Inc. (Santa Cruz, CA, USA). All of other chemicals were of the highest quality available from the commercial vendors.

### Tissue collection and cell isolation

The spleens were collected from the normal C57BL/6 mice, and the excess fat was removed. The T cells were dissociated from the spleens, and the cell suspension was washed. The gradient separation was performed to remove other cells. The cells were collected by the centrifugation and were resuspended in the RPMI‐1640 containing 10% foetal bovine serum for the investigation.

### Naïve CD4^+^ T cell purification and treatment

The naïve CD4^+^ T cells were isolated from the spleens by the paramagnetic bead enrichment according to the manufacturer's protocol. The splenocytes were incubated with anti‐CD4/CD62L‐coated magnetic beads and were isolated using MACS separation columns (Miltenyi, CA, USA). The cell purity more than 98% was confirmed using the flow cytometry assay. The naïve CD4^+^ T cells were treated with soluble anti‐CD3 (1 μg/ml) and anti‐CD28 (1 μg/ml) (BD Bioscience, CA, USA) for 72 hrs and were induced to differentiate into the Th17 cells with TGF‐β (2 ng/ml) and IL‐6 (20 ng/ml) in the presence of TCDD (5 nM) and tetrandrine (0.1, 0.3 and 1 μM). To investigate whether STAT3 and STAT5 are involved in the tetrandrine‐induced inhibition of the Th17 cell differentiation, the STAT3 activator colivelin (100 pM) 24 or the STAT5 inhibitor HPA (50 μM) 25 was co‐added with tetrandrine (1 μM) into the naïve CD4^+^ T cells. Furthermore, to investigate whether AhR is involved in the effect of tetrandrine, the AhR antagonist CH223191 (3 μM) was co‐added with tetrandrine (1 μM) into the naïve CD4^+^ T cells.

### Protein extractions

The pre‐treated CD4^+^ T cells were harvested and were lysed in the buffer: 1% Nonidet P‐40, 50 mM Tris‐HCl, 250 mM NaCl, 5 mM EDTA, 10 mM Na_3_VO_4_, 0.5 mM DTT and containing the protease inhibitor phenylmethanesulfonyl fluoride (1 mM). The homogenate was centrifuged for 10 min by 12,000 *g* at 4°C.

The cytoplasmic and nuclear proteins were extracted from the pre‐treated CD4^+^ T cells using the reagent kit (Vazyme, Nanjing, China) as previously described 26. The protein concentrations were determined using the bicinchoninic acid method with a commercial kit (Beyotime, Shanghai, China).

### Western blot assay

The proteins were loaded onto the 10% polyacrylamide gels and subjected to the sodium dodecyl sulphate polyacrylamide gel electrophoresis, and then transferred to the nitrocellulose membranes (Millipore, MA, USA). The membranes were blocked in 5% (w/v) non‐fat milk at 25°C and then incubated with mouse primary antibody overnight at 4°C. Then, the HRP‐conjugated secondary antibody was added. The hybridized bands were detected using the enhanced chemiluminescence reagent kit (Bioworld, St. Louis Park, MN, USA). These results were determined by the densitometric analysis (Image‐Pro Plus) and then corrected by the GAPDH.

### Immunofluorescence assay

The cells were fixed in the 4% paraformaldehyde and permeabilized in the 1% Triton X‐100 at 25°C. Then, the cells were blocked in the 10% normal goat serum and were incubated with mouse antibody against p‐STAT3 overnight at 4°C. The Rhodamine‐conjugated secondary antibody (Bioworld, Nanjing, China) was used to incubate the cells. The nucleus was stained with the DAPI (Bioworld, Nanjing, China) for the cell imaging. The fluorescence was visualized using an inverted microscope (Olympus, Japan).

### Flow cytometry

The pre‐treated CD4^+^ T cells were washed, and the following mouse antibodies were added for intracellular staining: anti‐CD4‐PE, anti‐IL‐17A‐FITC, anti‐CD25‐FITC and anti‐Foxp3‐APC (eBioscience, San Diego, CA, USA). The cells were fixed in the 4% paraformaldehyde and were permeabilized in the 1% Triton X‐100 at 25°C. The flow cytometry was performed on a FACS Canto™ II (BD, USA). The events were recorded and then analysed using the FlowJo7.6.1 software (Tree Star, Palo Alto, CA, USA).

### Quantitative PCR assay

The total RNAs were extracted from the pre‐treated CD4^+^ T cells with TRIzol Reagent (Invitrogen, Carlsbad, CA, USA). The extracted RNAs (1 pg–500 ng) were reversely transcribed into cDNAs using a high‐capacity cDNA synthesis kit (Vazyme, Nanjing, China). The mRNA expression was determined by the real‐time PCR detection system (Bio‐Rad, USA) using a SYBR Green PCR kit (Vazyme, Nanjing, China) and the mouse pairs of oligonucleotide primers in Table 1. The following conditions were used: 5 min at 95°C (1 cycle), 10 sec at 95°C and 30 sec at 60°C (40 cycles). The results were calculated to the GAPDH gene on the base of 2^−ΔΔCt^ algorithm.

**Table 1:** Mouse primer sequences used for the quantitative PCR assay

Gene	Forward primer (5′–3′)	Reverse primer (5′–3′)
Foxp3	ATGCCCAACCCTAGGCCAGCCAAG	TGGGCCCCACTTCGCAGGTCCCGAC
IL‐10	GCTATGTTGCCTGCTCTTACTG	TCTGGCTGACTGGGAAGTG
RORγt	TACCCTACTGAGGACAGG	CCACATTACACTGCTGGCTG
IL‐17A	TACCTCAACCGTTCCACGTC	TTTCCCAACCGCATTGACACA
GAPDH	GGTGAAGGTCGGTGTGAACG	CTCGCTCCTGGAAGATGGTG

### Transient transfection

The naïve CD4^+^ T cells were inoculated in 6‐well plate at a density of 1 × 10^6^ cells per well and then stimulated with anti‐CD3/CD28 and treated with TGF‐β and IL‐6. The siRNAs for the AhR (siAhR) and the STAT5a/b (siSTAT5), and the non‐target‐specific control siRNA (siCtrl, RiboBio, Guangzhou, China) were transfected into the cells by a transfection reagent Lipofectamine™ 2000 (Invitrogen, Carlsbad, USA). Thus, the cells were incubated with transfection complexes for 72 hrs in the presence or absence of tetrandrine. The total RNAs and proteins were extracted from the cells for further investigation.

### Co‐immunoprecipitation assay

The proteins were extracted from the pre‐treated CD4^+^ T cells which were homogenized in the lysis buffer. The 1 μg of mouse antibody against AhR (Santa Cruz, CA, USA) was added to the extracted proteins and was rotated overnight at 4°C. Then, the 20 μl of Protein A Dynabeads (Bioworld, MN, USA) was blended in the extracted proteins and then rotated overnight at 4°C. The beads were washed with the lysis buffer, and the immunopurified proteins were eluted by boiling the beads in the 40 μl loading buffer.

### Induction of CIA and administration of test samples

The male DBA/1 mice were immunized by an intradermal injection at the base of the tail with 100 μg chicken type II collagen (Sigma‐Aldrich) emulsified in Freund's complete adjuvant (Sigma‐Aldrich) as previously described 4. On day 21 after the first immunization, the mice were boosted in the same way using the collagen emulsified in Freund's incomplete adjuvant. When the mice showed visible clinical signs resulting in a clinical score of ≥3, treatment was started. Resveratrol and tetrandrine were equably suspended in 0.5% carboxymethylcellulose sodium (CMC‐Na). To investigate the role of AhR in the tetrandrine‐mediated regulation of the activities of STAT3 and STAT5 in the CIA mice, the resveratrol was orally administered at 20 mg/kg, and the tetrandrine was orally administered at 40 mg/kg per day for 14 consecutive days from the day 28 after the first immunization. The vehicle group was orally administered an equivalent volume of 0.5% CMC‐Na.

### Immunohistochemical assay

The immunohistochemical assay was performed as previously described 16. The spleens and mesenteric lymph nodes (MLNs) isolated from the CIA mice were fixed in the 4% paraformaldehyde and were embedded in the paraffin and then sectioned. The tissue slices were incubated with the mouse antibodies against p‐STAT3 and p‐STAT5 overnight and were incubated with a biotinylated secondary antibody and a streptavidin‐peroxidase complex for 1 h. The final coloured product was developed using the diaminobenzidine chromogen. Then, the slices were counterstained with the haematoxylin and then photographed using an inverted microscope (Olympus, Japan). The results were analysed using the Image‐Pro Plus software.

### Statistical analysis

The data were expressed as the means ± SD of the three independent experiments. Statistical analysis was performed using the SPSS version 17.0 software. The Student's *t*‐test was used to analyse matched values, and the one‐way ANOVA was used to analyse multiple mean values. A value of *P* < 0.05 was considered statistically significant.

## Results

### Effect of tetrandrine on the activation of JAK/STAT signalling pathway during the Th17 cell differentiation

**Figure 1:**
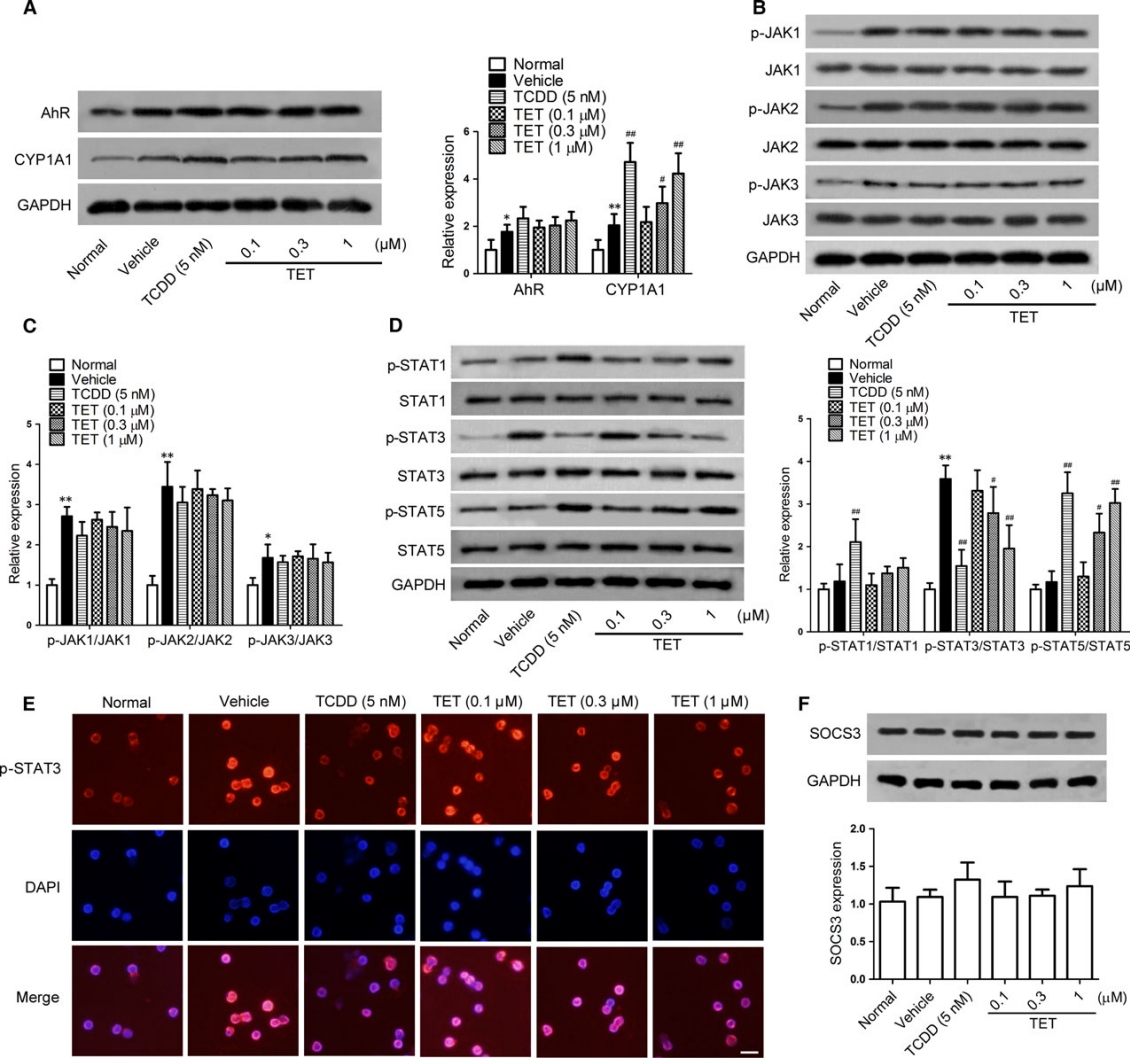
The effect of tetrandrine on the activation of JAK/STAT signalling pathways during the Th17 cell differentiation. The naïve CD4^+^ T cells were stimulated with anti‐CD3/CD28 and were treated with TGF‐β and IL‐6 in the presence or absence of TCDD and tetrandrine (TET) for 72 hrs. (**A**) The expression of AhR and CYP1A1 was determined using the Western blot assay, and the intensities of proteins were normalized to that of the GAPDH. (**B** and **C**) The protein levels of p‐JAK1, JAK1, p‐JAK2, JAK2, p‐JAK3 and JAK3 were determined using the Western blot assay, and the intensities of proteins were normalized to that of the GAPDH. (**D**) The protein levels of p‐STAT1, STAT1, p‐STAT3, STAT3, p‐STAT5 and STAT5 were determined using the Western blot assay, and the intensities of proteins were normalized to that of the GAPDH. (**E**) The effect of tetrandrine on the nuclear translocation of STAT3. The cells were fixed and then subjected immunofluorescence assay with mouse p‐STAT3 antibody (*upper column*), and the images were superimposed with DAPI‐stained images (*middle column*) and merged images (*lower column*). The representative photographs from the each group are shown. Scale bar, 50 μm. (**F**) The SOCS3 expression was determined using the Western blot assay, and the intensities of proteins were normalized to that of the GAPDH. The data are from the three independent experiments. *P < 0.05, **P < 0.01 *versus* the normal group; ^#^ *P* < 0.05, ^##^ *P* < 0.01 *versus* the vehicle‐treated group.

The JAK/STAT signalling pathways are closely linked to the Th17 cell differentiation. To explore how tetrandrine suppresses Th17 cell differentiation through the AhR pathway, the naïve CD4^+^ T cells were activated with anti‐CD3/CD28 under the Th17‐polarizing condition, and the phosphorylations of JAK and STAT were detected by the Western blot assay. Similar to TCDD, tetrandrine promoted the expression of the AhR target gene CYP1A1 at protein level, but did not affect the expression of AhR itself (Fig. 1A). Tetrandrine was lack of significant effect on the phosphorylation levels of JAK1, JAK2 and JAK3 (Fig. 1B and C). But it markedly inhibited the phosphorylation of STAT3 and boosted the phosphorylation of STAT5 without the effect on the phosphorylation of STAT1 (Fig. 1D). The nuclear translocation of p‐STAT3 was also inhibited by the both tetrandrine and TCDD (Fig. 1E). Moreover, tetrandrine did not significantly alter the expression of SOCS3, an intrinsic negative regulator of the JAK/STAT signalling pathways (Fig. 1F). Together, the results suggested that tetrandrine could inhibit the phosphorylation of STAT3 and enhance the phosphorylation of STAT5 under the Th17‐polarizing condition, and its functions were independent of the upstream JAK signallings.

### Involvements of STAT3 and STAT5 in tetrandrine‐induced inhibition of the Th17 cell differentiation

**Figure 2:**
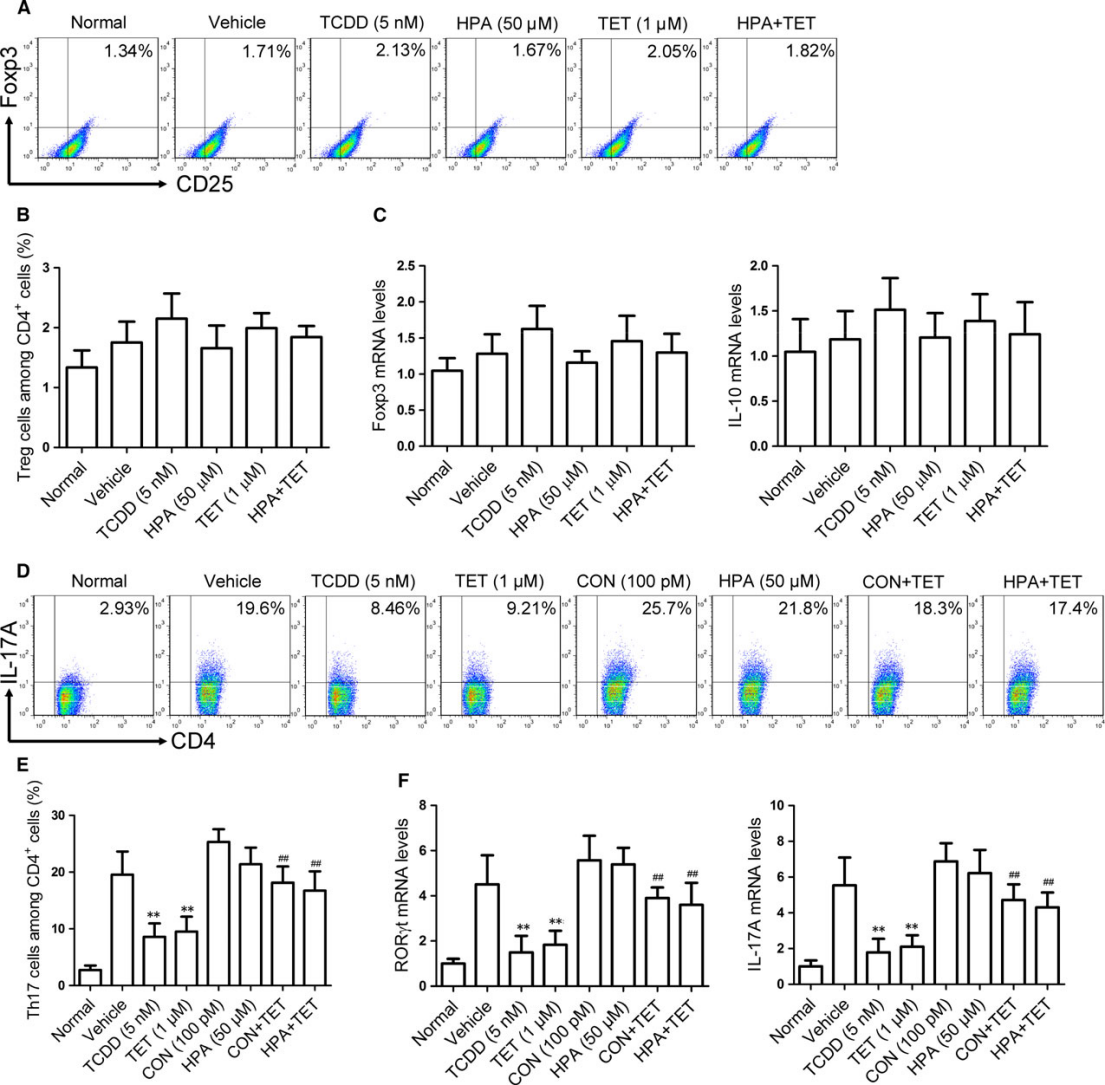
The relevance between the regulation of STAT3 and STAT5 activities and the inhibition of Th17 cell differentiation by the tetrandrine treatment during the Th17 cell differentiation. The effect of tetrandrine on the Treg cell differentiation during the Th17 cell differentiation was investigated. The naïve CD4^+^ T cells were stimulated with anti‐CD3/CD28 and were treated with TGF‐β and IL‐6 in the presence or absence of tetrandrine (TET) and HPA (an inhibitor of STAT5) for 72 hrs. (**A** and **B**) The frequency of Treg cells in the CD4^+^ T cells was determined using the flow cytometry assay. (**C**) The expression of the mRNAs for the transcription factor Foxp3 and the cytokine IL‐10 was determined using the quantitative PCR assay. The colivelin (CON, an activator of STAT3) or the HPA was added into the naïve CD4^+^ T cells together with tetrandrine for 72 hrs. (**D** and **E**) The frequency of Th17 cells in the CD4^+^ T cells was determined using the flow cytometry assay. (**F**) The expression of the mRNAs for the transcription factor RORγt and the cytokine IL‐17A was determined using the quantitative PCR assay. The data are from the three independent experiments. **P < 0.01 *versus* the vehicle‐treated group; ^##^ *P* < 0.01 *versus* the tetrandrine‐treated group.

Tetrandrine could markedly enhance the activation of STAT5, an important regulator for the differentiation of Treg cells. To rule out the possibility that tetrandrine‐mediated inhibition of the differentiation of Th17 cells was achieved by induction of the differentiation of Treg cells *via* STAT5, the naïve CD4^+^ T cells were stimulated with anti‐CD3/CD28 and treated with TGF‐β and IL‐6 in the presence or absence of tetrandrine and HPA (an inhibitor of STAT5), and the effect of tetrandrine on Treg cell differentiation under the Th17‐polarizing condition was observed. Tetrandrine and TCDD did not significantly affect the differentiation of Treg cells (Fig. 2A and B) and the expression of the mRNAs for the related transcription factor Foxp3 and the cytokine IL‐10 (Fig. 2C), suggesting that tetrandrine might directly inhibit the differentiation of Th17 cells.

To recognize the relevance between the regulation of STAT3 and STAT5 activities and the inhibition of Th17 cell differentiation by tetrandrine, the colivelin (an activator of STAT3) and the HPA were added into the naïve CD4^+^ T cells together with tetrandrine. The results showed that the tetrandrine‐mediated inhibition of the Th17 cell differentiation (Fig. 2D and E) and the expression of the mRNAs for the related transcription factor RORγt and the cytokine IL‐17A (Fig. 2F) was largely diminished by either colivelin or HPA. It was suggested that STAT3 and STAT5 were involved in the tetrandrine‐mediated inhibition of the Th17 cell differentiation.

### Role of AhR in the tetrandrine‐mediated regulation of the activities of STAT3 and STAT5

**Figure 3:**
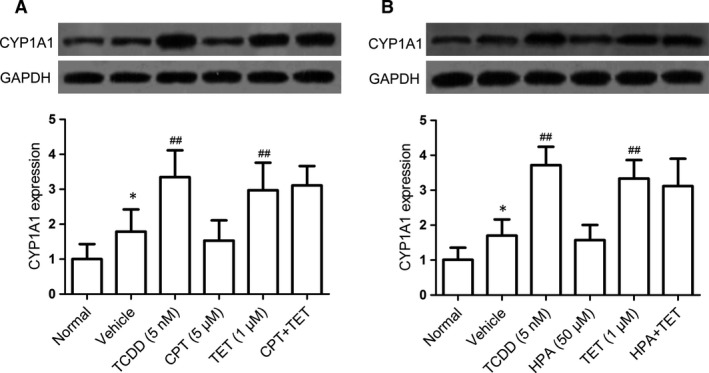
The involvements of STAT3 and STAT5 in the activation of AhR by the tetrandrine treatment during the Th17 cell differentiation. The naïve CD4^+^ T cells were treated with CPT (an inhibitor of STAT3) and HPA (an inhibitor of STAT5) in the presence or absence of tetrandrine (TET) for 72 hrs. The effects of CPT (**A**) and HPA (**B**) on the tetrandrine‐induced CYP1A1 expression were analysed using the Western blot assay, and the intensities of proteins were normalized to that of the GAPDH. The data are from the three independent experiments. *P < 0.05 *versus* the normal group; ^##^ *P* < 0.01 *versus* the vehicle‐treated group.

To now, little is known about the relation of STAT3 and STAT5 to the AhR. To recognize whether STAT3 and STAT5 are the upstream signallings for the activation of AhR induced by tetrandrine, the naïve CD4^+^ T cells were treated with the CPT (an inhibitor of STAT3) and the HPA in the presence or absence of tetrandrine under the Th17‐polarizing condition. As shown in Fig. 3A and B, tetrandrine promoted the expression of CYP1A1, and neither CPT nor HPA affected the effect of tetrandrine, which excluded the possibility that tetrandrine induced the activation of AhR through regulating the activities of STAT3 and STAT5.

**Figure 4:**
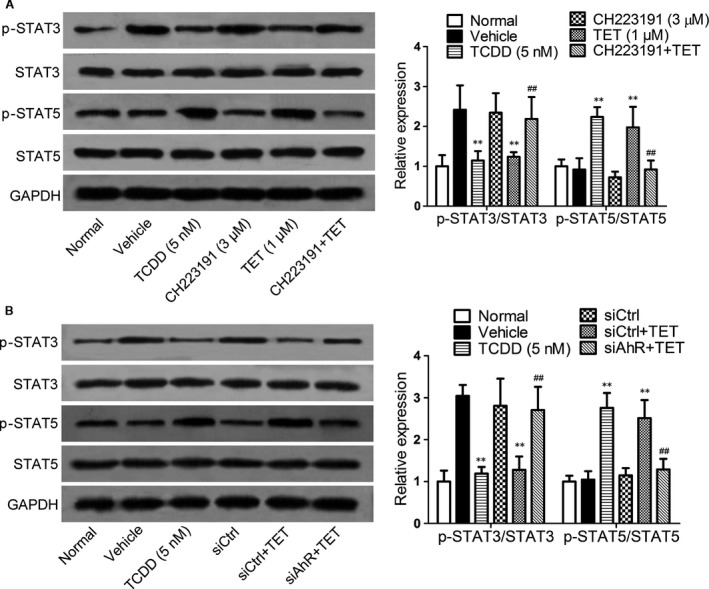
The involvement of AhR in the regulation of STAT3 and STAT5 activities by the tetrandrine treatment during the Th17 cell differentiation. The AhR antagonist CH223191 were added into the naïve CD4^+^ T cells in the presence or absence of tetrandrine (TET) for 72 hrs. (**A**) The effect of CH223191 on the regulation of STAT3 and STAT5 phosphorylations by the tetrandrine treatment was analysed using the Western blot assay. (**B**) The effect of AhR knockdown on the regulation of STAT3 and STAT5 phosphorylations by the tetrandrine treatment was analysed using the Western blot assay. The intensities of proteins were normalized to that of the GAPDH. The data are from the three independent experiments. **P < 0.01 *versus* the vehicle‐treated group; ^##^ *P* < 0.01 *versus* the tetrandrine‐treated group.

In contrast, we investigated whether the AhR was required for the tetrandrine‐mediated regulation of the activities of STAT3 and STAT5, the AhR antagonist CH223191 was added into the naïve CD4^+^ T cells in the presence or absence of tetrandrine under the Th17‐polarizing condition. Similar to TCDD, tetrandrine inhibited the phosphorylation of STAT3 and enhanced the phosphorylation of STAT5, which were almost completely reversed by CH223191 (Fig. 4A). Furthermore, the AhR knockdown reversed the decrease in STAT3 phosphorylation and the increase in STAT5 phosphorylation induced by the tetrandrine treatment (Fig. 4B). These results indicated that AhR played a crucial role in the tetrandrine‐mediated regulation of the activities of STAT3 and STAT5.

### *In vivo* verification of the involvement of AhR in the tetrandrine‐mediated regulation of the activities of STAT3 and STAT5

**Figure 5:**
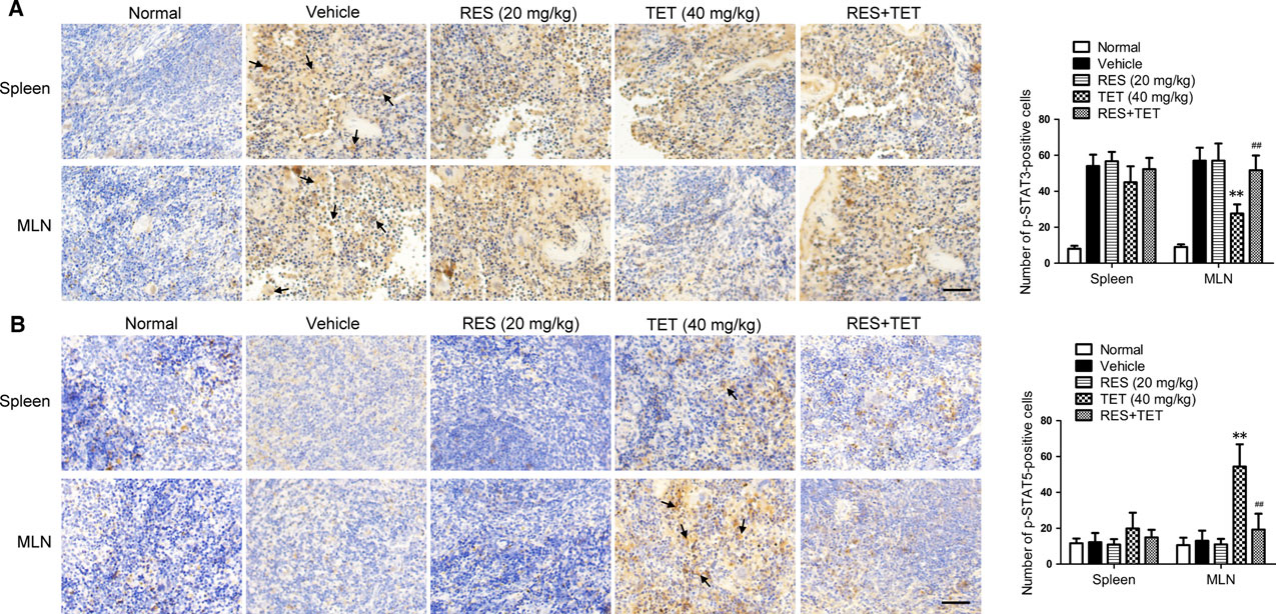
*In vivo* verification of the involvement of AhR in the regulation of STAT3 and STAT5 phosphorylations by the tetrandrine treatment. The male DBA/1 mice were immunized with the type II collagen; the AhR antagonist resveratrol (RES) and tetrandrine (TET) were orally co‐administered for 14 consecutive days from the day 28 after the first immunization. When the mice were sacrificed, spleens and MLNs were taken for the examination of the expression of p‐STAT3 and p‐STAT5. The phosphorylation levels of STAT3 (**A**) and STAT5 (**B**) in spleens and MLNs were determined using the immunohistochemical assay. Scale bar, 50 μm. The Image‐Pro Plus software was performed to analyse the positive cells that express p‐STAT3 and p‐STAT5 per the high power field and the mean density. The representative photographs from each group are shown (*n* = 6 mice per group). **P < 0.01 *versus* the vehicle‐treated group; ^##^ *P* < 0.01 *versus* the tetrandrine‐treated group.

In the CIA mice, the relevance between the activation of AhR and the regulation of STAT3 and STAT5 activities by the tetrandrine treatment was verified. The resveratrol (an antagonist of AhR) was orally administered together with tetrandrine for 14 consecutive days from the day 28 after the first immunization of type II collagen. When the mice were sacrificed, the spleens and the MLNs were taken for the examination of p‐STAT3 and p‐STAT5 expression. Figure 5A showed that p‐STAT3 levels were markedly increased in the vehicle‐treated mice. The resveratrol did not significantly affect the STAT3 phosphorylation. However, tetrandrine could downregulate p‐STAT3 levels in the MLNs, which was almost completely reversed by resveratrol. Figure 5B showed that p‐STAT5 levels were not altered in the vehicle‐treated mice and resveratrol‐treated mice. Tetrandrine markedly up‐regulated p‐STAT5 levels in the MLNs, which was also largely diminished by resveratrol. It was suggested that AhR was required for the regulation of tetrandrine on STAT3 and STAT5 activities in CIA mice.

### The role of AhR for the tetrandrine‐mediated regulation of STAT3 and STAT5 activities

**Figure 6:**
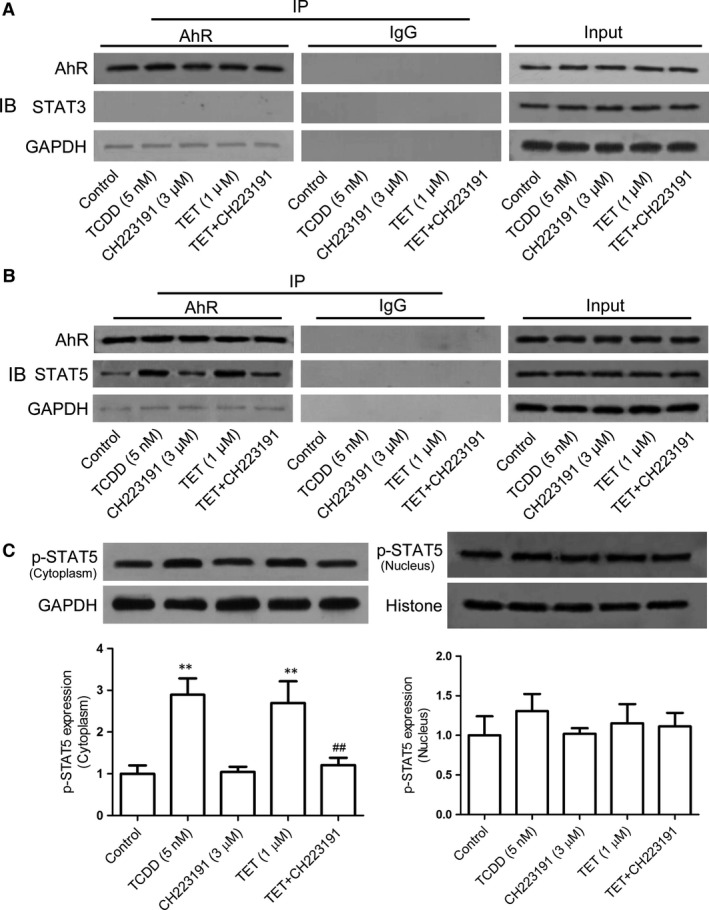
The role of AhR for the STAT3 and STAT5 by the tetrandrine treatment during the Th17 cell differentiation. The naïve CD4^+^ T cells were stimulated with anti‐CD3/CD28 and were treated with TGF‐β and IL‐6 in the presence or absence of tetrandrine (TET) and AhR antagonist CH223191 for 72 hrs. The role of AhR for the STAT3 (**A**) and STAT5 (**B**) was analysed using the co‐immunoprecipitation assay. (**C**) The expression of p‐STAT5 in the cytoplasm and the nucleus was determined using the Western blot assay, and the intensities of proteins were normalized to that of the internal controls GAPDH and Histone. The data are from the three independent experiments. **P < 0.01 *versus* the control group; ^##^ *P* < 0.01 *versus* the tetrandrine‐treated group.

To further explore how the activation of AhR by tetrandrine regulates the activities of STAT3 and STAT5, the naïve CD4^+^ T cells were activated with anti‐CD3/CD28 in the presence or absence of tetrandrine and AhR antagonist CH223191 under the Th17‐polarizing condition, and the co‐immunoprecipitation assay was performed to analyse the interaction between AhR, STAT3 and STAT5. Similar to TCDD, tetrandrine could not induce the binding of the AhR to the STAT3 protein, but it promoted the AhR directly binding to the STAT5 protein, which could be reversed by CH223191 (Fig. 6A and B). Tetrandrine and TCDD also increased STAT5 phosphorylation levels in the cytoplasm but not in the nucleus (Fig. 6C). The results suggested that tetrandrine could induce a direct binding of AhR to the STAT5 protein and promote the phosphorylation of the latter.

### The relevance between the decreased activation of STAT3 and the increased activation of STAT5 induced by tetrandrine

**Figure 7:**
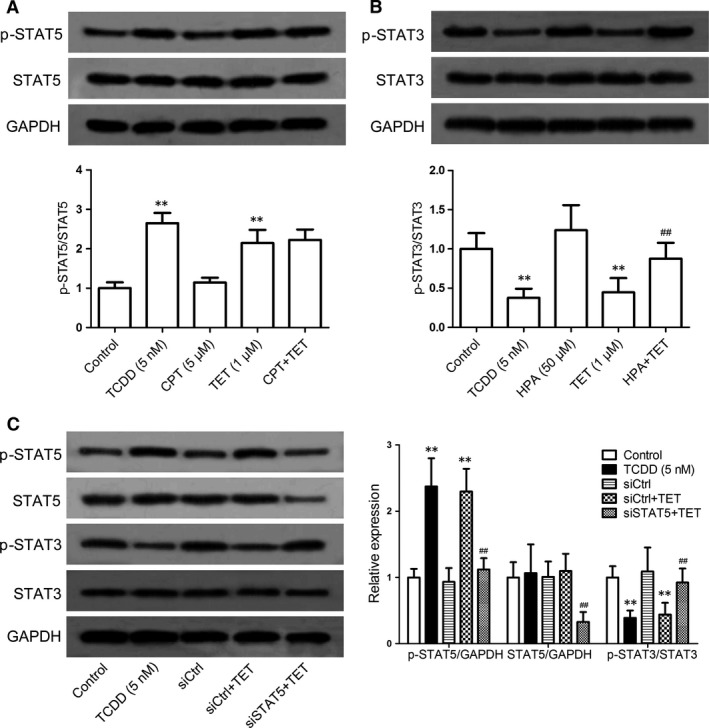
The relevance between the decreased STAT3 phosphorylation and the increased STAT5 phosphorylation by the tetrandrine treatment during the Th17 cell differentiation. The naïve CD4^+^ T cells were treated with CPT (an inhibitor of STAT3) and HPA (an inhibitor of STAT5) in the presence or absence of tetrandrine (TET) for 72 hrs. (**A**) The involvement of STAT3 in the promotion of STAT5 phosphorylation by the tetrandrine treatment was analysed using the Western blot assay. (**B**) The involvement of STAT5 in the inhibition of STAT3 phosphorylation by the tetrandrine treatment was analysed using the Western blot assay. (**C**) The effect of STAT5 knockdown on the promotion of STAT5 phosphorylation and the inhibition of STAT3 phosphorylation by the tetrandrine treatment was analysed using the Western blot assay. The intensities of proteins were normalized to that of the GAPDH. The data are from the three independent experiments. **P < 0.01 *versus* the control group; ^##^ *P* < 0.01 *versus* the tetrandrine‐treated group.

To recognize the relevance between the inhibition of STAT3 activity and the promotion of STAT5 activity by the tetrandrine treatment, CPT and HPA were added into the naïve CD4^+^ T cells in the presence or absence of tetrandrine under the Th17‐polarizing condition for 72 hrs. It was shown that CPT was unable to affect the tetrandrine‐mediated phosphorylation of the STAT5 (Fig. 7A), but the HPA markedly diminished the tetrandrine‐induced inhibition of the phosphorylation of STAT3 (Fig. 7B). Meanwhile, the knockdown of STAT5 almost completely reversed the increase in STAT5 phosphorylation and the decrease in STAT3 phosphorylation induced by tetrandrine (Fig. 7C). It was suggested that tetrandrine suppressed the activation of STAT3 through the STAT5 pathway.

## Discussion

The AhR may play an important regulatory role in the T cell differentiation and thereby ameliorate CIA in mice 4, 27. The activation of AhR by its high‐affinity ligand TCDD exerts potent immunosuppressive effects in the T cell‐associated diseases. The TCDD‐mediated inhibitory effect occurs early during the CD4^+^ T cell differentiation and inhibits Th17 cell differentiation 28. Moreover, the optimal inhibition of the cytotoxic T lymphocyte response takes place when TCDD is added within 3 days of alloantigen stimulation during the differentiation of CD4^+^ T cells 29.

Our previous studies have shown that tetrandrine can ameliorate mouse CIA by suppressing Th17 cell differentiation in a manner dependent of the AhR pathway. The relative activation of STAT3 and STAT5 is closely linked to the Th17 cell differentiation. In this study, tetrandrine and TCDD decreased the STAT3 activity and enhanced the STAT5 activity under the Th17‐polarizing condition, while did not alter the activities of JAK1, JAK2 and JAK3. Meanwhile, tetrandrine and TCDD suppressed the nuclear translocation of the activated STAT3. It is well known that STAT3 plays a pivotal role in the differentiation of Th17 cells and is involved in the development of autoimmune diseases 30. The IL‐6 signalling in concert with TGF‐β initiates RORγt expression through the STAT3 activation, which leads to promotion of the expression of proinflammatory cytokines 31, 32. In contrast, STAT5 functions as a negative regulator in the differentiation of Th17 cells 19. The loss of STAT5 in the CD4^+^ T cells results in the development of autoimmune diseases which is associated with the overproduction of the cytokine IL‐17 33, 34. The balance between STAT3 and STAT5 signallings rather than their absolute levels is more important for the Th17 cell differentiation 17. In addition, the SOCS3 suppresses the activity of cytokine receptors by interacting with the JAK as a pseudosubstrate and inhibiting its kinase activity and displays an important role in constraining IL‐17 production 35, 36. In the present study, the expression of SOCS3 was not altered by the tetrandrine treatment. It was suggested that tetrandrine modulated the activities of STAT3 and STAT5 not by the JAK pathways.

The activation of STAT3 can negatively modulate the differentiation of Treg cells, while the activation of STAT5 appears to be essential for the Treg cell differentiation and sustaining of the expression of the transcription factor Foxp3 37, 38. Of interest, the differentiation of Treg cells was not changed by tetrandrine treatment under the Th17‐polarizing conditions, suggesting that activation of STAT5 induced by tetrandrine was little relative to the Treg cell differentiation. The STAT3 activation can suppress the binding of the activated STAT5 to the Foxp3 promoter gene, significantly more activated STAT5 binds to the region of Foxp3 locus when STAT3 is absent 39. Tetrandrine inhibited Th17 cell differentiation through the inhibition of STAT3 activation and the promotion of STAT5 activation.

Whatever, the expression of AhR is robustly initiated under the Th17‐polarizing condition 21. TCDD‐activated AhR can suppress the production of IL‐17 and the expression of RORγt in the CD4^+^ T cells 22. Furthermore, the AhR interacts with the STAT proteins and modulates their activities 40. Tetrandrine was shown to regulate the activities of STAT3 and STAT5 through the AhR pathway. The activation of AhR by the tetrandrine treatment negatively regulated the activation of STAT3 and the expression of RORγt induced by IL‐6 and TGF‐β, while positively regulated the activation of STAT5.

Recent studies have shown that the decreased STAT3 activity can attenuate the autoimmune arthritis through reducing the proportion of Th17 cells 41, while the increased STAT5 activity can ameliorate mouse CIA 42. Thus, the *in vivo* relevance between the regulation of STAT3 and STAT5 activities and the activation of AhR by the tetrandrine treatment was further investigated. In CIA mice, tetrandrine inhibited STAT3 activity and enhanced STAT5 activity through the AhR pathway in the MLNs. Of note, MLNs, a crossroad between the mucosal and the peripheral recirculation pathways, have an important role in the immune modulation and the prevention of autoimmunity 43, 44.

Concerning on the interaction between the activated AhR and the proteins of STAT3 and STAT5, tetrandrine enhanced the AhR binding to the STAT5 protein similar to TCDD, but not to the STAT3 protein. It was suggested that the activation of AhR by tetrandrine might directly enhance STAT5 activity, which negatively regulated STAT3 activity. Moreover, tetrandrine enhanced the combination of AhR with STAT5 to form a complex, which might account for the promotion of STAT5 activity.

Finally, the relevance between the decreased STAT3 activity and the increased STAT5 activity by tetrandrine was further explored. As a negative regulator in the STAT family, the STAT5 can inhibit STAT3 activation. Tetrandrine suppressed STAT3 activity *via* the STAT5 activation. Recent studies have focused on the reciprocal functions of STAT3 and STAT5 in T cells, and it appears that relative activations of STAT3 and STAT5 regulate the balance between Th17 and Treg cells 17, 45. The differentiation of Th17 cells is inhibited by the IL‐2 signalling through the induction of STAT5 activity 34. Thus, the promoted STAT5 activity by tetrandrine *via* the AhR pathway might lead to an inhibitory effect on the STAT3 activation.

In conclusion, tetrandrine decreased STAT3 activity and increased STAT5 activity to inhibit Th17 cell differentiation through the AhR pathway. The findings were helpful for understanding the anti‐CIA mechanism of tetrandrine in more detail.

## Conflict of interests

The authors confirm that there are no conflict of interests.

## Author contribution

X. Yuan, Z. Wei and Y. Dai designed the studies; X. Yuan, Y. Dou and X. Wu performed the researches; X. Yuan and Y. Dai analysed the data and wrote the manuscript. All authors reviewed the study.
